# Assessing Water Filtration and Safe Storage in Households with Young Children of HIV-Positive Mothers: A Randomized, Controlled Trial in Zambia

**DOI:** 10.1371/journal.pone.0046548

**Published:** 2012-10-17

**Authors:** Rachel Peletz, Martin Simunyama, Kelvin Sarenje, Kathy Baisley, Suzanne Filteau, Paul Kelly, Thomas Clasen

**Affiliations:** 1 London School of Hygiene and Tropical Medicine, London, United Kingdom; 2 Tropical Gastroenterology and Nutrition Group, University Teaching Hospital, Lusaka, Zambia; 3 Barts and The London School of Medicine, Queen Mary, University of London, London, United Kingdom; University of Ottawa, Canada

## Abstract

**Background:**

Unsafe drinking water presents a particular threat to people living with HIV/AIDS (PLHIV) due to the increased risk of opportunistic infections, diarrhea-associated malabsorption of essential nutrients, and increased exposure to untreated water for children of HIV-positive mothers who use replacement feeding to reduce the risk of HIV transmission. This population may particularly benefit from an intervention to improve water quality in the home.

**Methods and Findings:**

We conducted a 12-month randomized, controlled field trial in Zambia among 120 households with children <2 years (100 with HIV-positive mothers and 20 with HIV-negative mothers to reduce stigma of participation) to assess a high-performance water filter and jerry cans for safe storage. Households were followed up monthly to assess use, drinking water quality (thermotolerant coliforms (TTC), an indicator of fecal contamination) and reported diarrhea (7-day recall) among children <2 years and all members of the household. Because previous attempts to blind the filter have been unsuccessful, we also assessed weight-for-age Z-scores (WAZ) as an objective measure of diarrhea impact. Filter use was high, with 96% (596/620) of household visits meeting the criteria for users. The quality of water stored in intervention households was significantly better than in control households (3 vs. 181 TTC/100 mL, respectively, *p*<0.001). The intervention was associated with reductions in the longitudinal prevalence of reported diarrhea of 53% among children <2 years (LPR = 0.47, 95% CI: 0.30–0.73, *p* = 0.001) and 54% among all household members (LPR = 0.46, 95% CI: 0.30–0.70, *p*<0.001). While reduced WAZ was associated with reported diarrhea (−0.26; 95% CI: −0.37 to −0.14, *p*<0.001), there was no difference in WAZ between intervention and control groups.

**Conclusion:**

In this population living with HIV/AIDS, a water filter combined with safe storage was used correctly and consistently, was highly effective in improving drinking water quality, and was protective against diarrhea.

**Trial Registration:**

Clinicaltrials.gov NCT01116908

## Introduction

Unsafe drinking water is a major cause of diarrheal death and disease, especially for young children in low-income countries and people living with HIV/AIDS (PLHIV). The 33 million PLHIV worldwide - including almost 1 million living in Zambia [1] - are especially vulnerable to diarrheal disease caused by opportunistic infections from waterborne pathogens, such as *Cryptosporidium* spp. [2], [3]. Diarrheal disease may lead to intestinal malabsorption so that PLHIV on antiretrovirals (ARVs) are not acquiring their essential nutrients and therapeutic dosages of medications [4], [5], [6].

Furthermore, diarrheal disease and unsafe drinking water may be particularly debilitating for children born to HIV-positive mothers. Young children born to HIV-positive mothers are at greater risk of mortality, morbidity, and malnutrition, which may be aggravated by enteric infection [7], [8], [9]. Safe water is critical for HIV-positive mothers who choose to replacement feed in order to prevent transmission of the virus via breast milk; “safe water and sanitation” is the first condition for replacement feeding in the new World Health Organization (WHO) guidelines [10]. Current WHO guidelines for infant feeding for HIV-positive women recommend that virtually all women breastfeed their children for up to 2 years while either the mother or child is on ARVs [10]; the risks of diarrheal disease and malnutrition outweigh the risks of HIV transmission in the majority of low-income settings. Even for mothers who choose to breastfeed, infants may be exposed to waterborne pathogens; exclusive breastfeeding is less common among HIV-positive mothers [11] and water treatment has been found to reduce diarrhea even among breastfed children [12]. Finally, young children who do contract the HIV virus will be more susceptible to water-related pathogens because of a weakened immune system and may particularly benefit from improved environmental conditions.

Our previous research in Zambia found that children <2 years born to HIV-positive mothers are particularly at risk of diarrheal disease. In our cross-sectional study, 26% of children <2 years had diarrhea in the past week and bacterial contamination of drinking water was found in 70% of households [13]. Children were more likely to have diarrhea if they had been given water in the past two days, suggesting that diarrheal disease may be at least partially attributable to unsafe drinking water. Additionally, diarrhea in children was significantly associated with mother's diarrhea, which is of particular concern in HIV-affected areas; mothers with HIV may be more likely to have diarrhea [2] and consequently more likely to pass diarrhea onto their children. Therefore, for children born to HIV-positive mothers in low-income settings, water quality interventions may be particularly critical.

Improving household drinking water quality through household water treatment and safe storage (HWTS) has been shown to have the potential to significantly reduce diarrheal disease [14], [15], [16]. International organizations including USAID, the World Bank, and WHO have recently called for an integration of water and sanitation activities in HIV/AIDS programs [17], [18], [19], [20], and the number of programs including HWTS for PLHIV is increasing [21], [22], [23], [24], [25], [26].

However, despite these programs, there is relatively little evidence demonstrating the health impact or examining use of HWTS interventions for PLHIV. Only one study has assessed the health impact of HWTS for PLHIV in a low-income setting in the form of a randomized, controlled trial. This trial in Uganda found that PLHIV with a household chlorination technology had 25% fewer diarrhea episodes and 33% fewer days with diarrhea compared to the control group, though diarrhea reductions were not significant for children under five [27]. Other observational studies of household chlorination interventions have found significant associations with diarrhea reductions in Nigeria among adults with HIV/AIDS [28] and in Kenya among infants born to HIV-positive mothers [29]. However, these studies and the majority of HWTS programs for PLHIV have been in the form of chlorination products [30], [31], [32], which do not inactivate or remove the full array of waterborne pathogens (such as *Cryptosporidium* spp.) unless combined with other treatment mechanisms [33]. Furthermore, there are questions about whether HWTS interventions are used correctly and consistently over an extended period of time [34], [35]; this study is primarily designed to examine HWTS use, which is vital to the success of HWTS programs.

We undertook a randomized controlled trial to assess a gravity water filter combined with local jerry cans for safe storage. Specifically, we examined 1) the use of the HWTS, both for children <2 years and all household members, 2) the microbiological performance of the HWTS intervention, measured as thermotolerant (fecal) coliforms (TTC), a well-established WHO indicator organism for fecal contamination [36], and 3) the impact of the intervention on the longitudinal prevalence of diarrhea among children <2 years and all household members, measured both as reported by the primary caretaker and by the weight-for-age z-score (WAZ) of children <2 years —a potential measure for reported diarrhea [37].

## Methods

The protocol for this trial and supporting CONSORT checklist are available as supporting information; see Checklist S1 and Protocol S1.

### Study Design and Sample Size

A randomized, controlled trial was designed to assess use, microbiological performance, and health impact of a household filtration intervention over 1 year. This study followed an open (non-blinded) design because previous attempts to blind the same intervention (LifeStraw Family filter) in the Congo were unsuccessful; the “placebo” provided to control households removed approximately 1 log (90%) of fecal contamination, potentially due to the formation of a biofilm, and the authors concluded that blinding this filter is not likely to be possible [38]. We estimated a sample size of 50 households per arm (100 total) would allow us to estimate use with a precision of at least ±15% with 20% loss to follow-up, assuming at least 70% use [38]. Additionally, 10 HIV-negative mothers and their households were included in each arm (20 total, an additional 20% of households). This figure represents a balance between the need to reduce potential stigma of participation and the cost and inconvenience to additional participants. Because recruitment occurred over an eight month period, the length of possible follow-up depended on the time of enrollment, up to 12 months. With this sample size, we had 80% power to detect a 40% reduction in diarrhea prevalence.

### Study Location

From our previous work [13], Chongwe District, Zambia was identified for this study based on the lack of piped water supply systems, inadequate water quality, and presence of active health clinics. The project sites included two neighboring compounds, Kasisi and Ngwerere in Chongwe District, both approximately 30 min–1 hour from central Lusaka, Zambia. Neither Kasisi nor Ngwerere were serviced by municipal piped water systems at the time of this study.

### Participant Eligibility and Enrollment

Children <2 years born to eligible HIV-positive mothers were targeted by recruiting and enrolling their mothers. Women were eligible to participate in the trial if they (i) had a child aged 6 to 12 months at the beginning of the trial, (ii) reported that they were HIV-positive (or HIV-negative) confirmed with antenatal clinic records and willing to disclose their status to our study team, and (iii) resided in a household located within the catchment areas of the Ngwerere or Kasisi health clinics in Chongwe district, Zambia and did not plan to move in the next 12 months. Health clinic staff identified potentially eligible women consecutively through under-five clinics and ART programs at their respective health clinics and referred them to our field team. HIV status of the children <2 years was recorded as reported by the mother.

### Intervention

Each intervention household received one LifeStraw Family filter and two 5-L safe storage containers. The LifeStraw Family is a novel HWTS filtration technology developed by Vestergaard-Frandsen SA that uses ultrafiltration in the form of a hollow-fiber cartridge to remove pathogens from drinking water [39]. To operate, untreated (influent) water is poured into a 2.5 L container, flows down a 1 m long tube designed to provide head pressure, and through the ulrafiltration cartridge where is it dispensed via tap (effluent). In addition to the filter, we provided two locally-procured 5-L jerry cans (Merco Ltd, Ndola, Zambia) for safely storing water following treatment. Households that were allocated to the intervention group received the filter and training on use and maintenance by our fieldworkers, who were previously trained by the filter manufacturer. Households allocated to the control group were instructed to continue usual practices throughout the study and were allocated filters and storage containers with subsequent training at the end of the study in August 2011.

### Baseline survey and randomization

At enrollment, baseline data were collected on demographics, sanitation facilities, hygiene practices, water sources and treatment practices, and feeding practices for children <2 years. For each household, baseline water samples were collected from drinking water sources and stored drinking water in the home. Households were randomly allocated using a computer random number generator to either a) the intervention group receiving the LifeStraw Family filter and storage containers, or b) the control group. The randomization was stratified by maternal HIV-status and catchment area (either Ngwerere or Kasisi) in blocks of 8 maximum. The randomization was conducted by the trial manager (RP) who was not involved in the enrollment of participants, and fieldworkers were not involved in the randomization.

Participants were recruited from April–December 2010, and followed for 7–12 months depending on time of recruitment. Households were considered to have completed the trial that continued until July 2011, regardless of the time of recruitment; total possible follow-up visits were calculated based on the time from enrollment until July 2011. Households were visited monthly; visits were unannounced and the field team made a repeat visit if the mother was not at home. Although we cannot rule out the potential of courtesy bias assessments of compliance, we took steps to minimize this by making all visits unannounced and sampling water quality, an objective measure.

### Outcome Measures

#### Use

Households were followed monthly to obtain information on filter use and acceptability. Households were classified as “reported users” if 1) the filter was observed in household at the time of visit, 2) the storage vessel contained water reported to be treated at the time of visit, and 3) the respondent reported using the filter on the day of or day prior to the day of visit. Households were classified as “confirmed users” if, in addition to these three criteria, there was at least a 1 log_10_ TTC improvement in their stored household water over their unfiltered water, or stored water quality was <10 TTC/100 mL. “Exclusive use” was defined as not drinking any unfiltered water in the day of or day prior to the interview as reported by the mother. The acceptability of the technology was evaluated through monthly household surveys.

#### Water Quality

Water samples were collected during monthly visits. For the stored drinking water, the respondent was asked if there was any drinking water in the house and samples were collected from the vessel that the householder identified for drinking. For control households, only stored drinking water was collected. For intervention households, water samples were collected of i) unfiltered water stored in the home (influent water), ii) filtered water immediately after filtration (effluent water), and iii) stored water that the household reported to be filtered, if available. Samples (125-mL) were collected in sterile Whirl-Pak™ Bags (Nasco International, Fort Atikinson, WI, USA) containing a tablet of sodium thiosulfate to neutralize any disinfectant, placed on ice, and processed within 4 hours of collection to assess levels of TTC/100 mL at the University Teaching Hospital, Zambia. Microbiological assessment was performed using a membrane filtration method with membrane lauryl suphate medium using using a DelAgua field incubator (Robens Institute, University of Surrey, Guildford, Surry, UK) in accordance with the Standard Methods [40]. After piloting the assay procedures, we elected to use full 100 ml samples for filtered and filtered & stored samples (intervention households) and 10-fold diluted samples for unfiltered samples (intervention and control households) to minimize the number of samples that yielded plates with colonies that were too numerous to count (TNTC). Where plates were TNTC, we ascribed a value of 500 TTC to such plates; this is a conservative estimate of the upper detection limit considering up to 1500 TTC were counted per plate. Baseline samples were also tested for free and total chlorine residuals using a Hach color-wheel test kit (Hach Company, Loveland, CO, USA).

#### Diarrhea Longitudinal Prevalence

At all monthly visits, the mother was asked whether each household member experienced any diarrhea in the past 7 days. Diarrhea was measured as longitudinal prevalence (the proportion of weeks with diarrhea divided by the number of weeks under observation) [41]. Diarrhea was defined according to the WHO definition of 3 or more loose stools within a 24-hour period [42], [43]. Mothers who reported diarrhea were also asked whether the episode extended for 14 days or longer in order to assess persistent diarrhea.

#### Weight-for-Age Z-scores (WAZ)

Children <2 years were weighed during monthly visits on baby scales (Seca Model 384, Chasmors, London, UK) according to standard protocol [44]. During weight measurements, children were only wearing a minimum of light clothing without shoes. Children were weighed a minimum of twice during every visit to verify the weight measurement; if the two measurements were not equal (particularly from child movement), the child was weighed a third time and the confirmed weight was recorded. Date of birth was verified on the child's health card to calculate WAZ.

### Data management and analysis

Data were double-entered into EpiData 3.1 and analyzed using Stata 12. The analysis plan was finalized before the data were examined. WAZ scores were calculated using the WHO growth reference data. Socioeconomic status was measured using an asset index created by combining data on household possessions and characteristics based on asset questionnaires used in the Zambia Demographic and Health Survey [45]. Data were analyzed on an intention-to-treat basis in order to estimate the effectiveness of supplying households with the intervention, regardless of filter use. The data from households with HIV-negative mothers were included in all analyses unless stated otherwise.

To assess acceptability and filter use, we tabulated data for all visits combined, and separately for the ‘final’ visit, defined as the final follow-up visit for households that completed the trial. To assess the effect of the intervention on water quality, TTC counts during follow-up were compared using random effects linear regression to account for repeated observations within households. TTC counts were normalized with log_10_ transformations; a value of 1 was added to all TTC levels before transformation to account for samples with TTC values of zero, log_10_(TTC level+1). Microbiological filter performance was calculated as the difference of the log of the influent concentration and log of the effluent concentration. All water quality analyses assumed that intervention households were drinking unfiltered water if stored filtered water was not available at the time of visit.

The effect of the intervention on diarrhea longitudinal prevalence was examined using binomial regression with a log link function and robust standard errors, with generalized estimating equations (GEE) to account for correlation of repeated measures within individuals [46]. In the analysis of diarrhea for all household members, we accounted for clustering at the household level, since this adequately accounted for within-individual correlation.

The effect of the intervention on WAZ was assessed using random effects linear regression to account for repeated observations within individuals. In a secondary analysis we controlled for WAZ at baseline. To examine the relationship between WAZ and diarrhea, we used random effects linear regression to account for repeated measures and adjusted for baseline WAZ.

To assess the relationship between water quality and diarrhea longitudinal prevalence, we used binomial regression with a log link function and robust standard errors with GEE to account for correlation of repeated measures. Water quality results were transformed to log_10_(TTC level+1), to account for samples with TTC values of zero. Adjusted analyses controlled for age and trial arm, since both were strongly associated with diarrhea. Predicted probabilities of diarrhea from the unadjusted and adjusted models were calculated at fixed values of log10 TTC and plotted.

We used fractional polynomials to examine the shape of the relationship of water quality (log_10_ TTC) with log diarrhea prevalence, using a set of defined powers (−2, −1, −0.5, 0.5, 1, 2 and ln(x)) and a maximum of two power terms in the model. Models were adjusted for intervention arm. The differences in model deviances were compared; the linear model was used if the improvement in fit was not statistically significant at p<0.05.

The relationship between water quality and WAZ was assessed with random effects linear regression accounting for repeated measures and adjusted for baseline WAZ; adjusting for baseline WAZ accounts for genetic variability and events prior to the intervention. To examine the effect of the intervention on mortality, we used a Cox Proportional hazard model to estimate mortality rates.

### Ethics

This study was approved by the Biomedical Research Ethics Committee of the University of Zambia and the London School of Hygiene and Tropical Medicine Ethics Committee, and registered with clinicaltrials.gov (NCT01116908). Participants were provided with verbal and printed details of the study in the local language; informed, written consent was obtained from all participating mothers for their respective households. Measures were taken to ensure confidentiality for all participants. If members of participating households were found to be in need of health care during the household visits, they were referred to health clinics. At the conclusion of the study, the results were disseminated to all participants in community meetings, and all control households received the intervention. Besides the intervention, households were not given incentives to participate.

## Results

### Study Population

**Figure 1:**
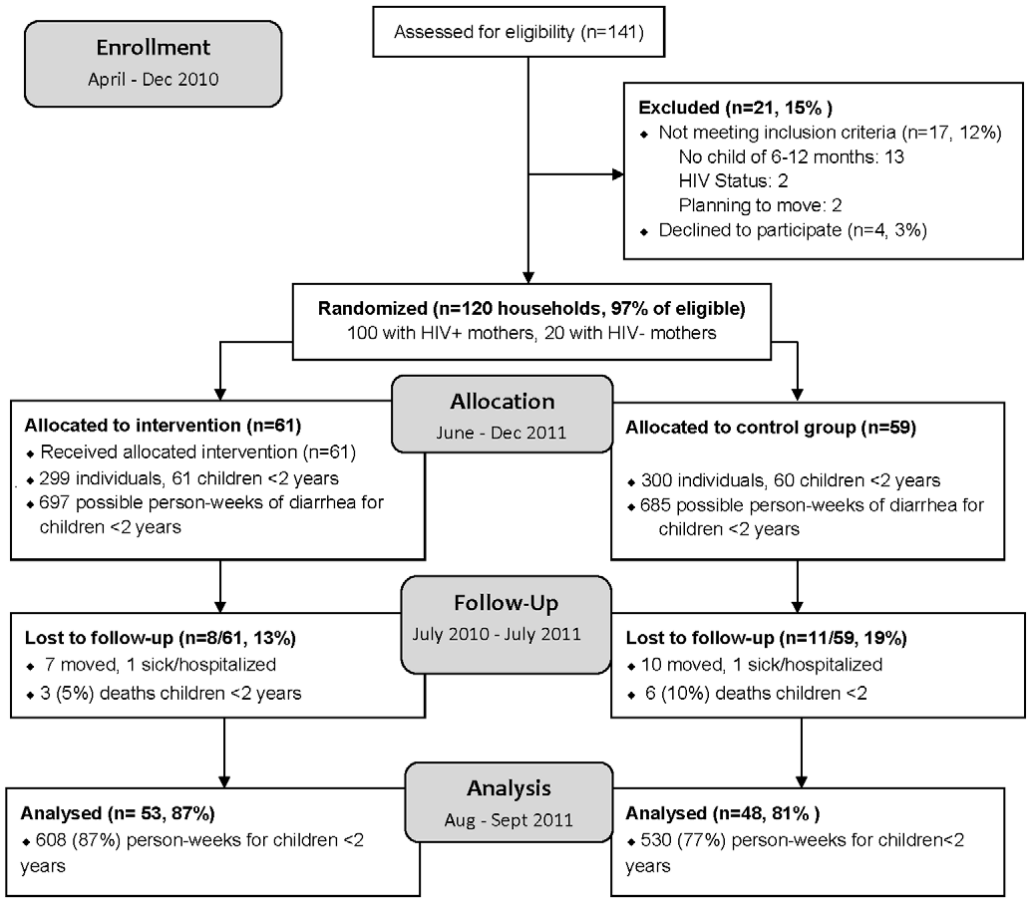
Recruitment flow diagram.

141 mothers were screened; 17 (12%) were ineligible and 4 (3%) refused to participate (Figure 1). Of the 120 households enrolled, 59 (49%) were allocated to the control group and 61 (51%) households were allocated to the intervention arm. One household in the control arm had twins; a total of 121 children <2 years were included. 91/120 (76%) households were enrolled for 12 months, the remaining were enrolled for 7–11 months; 101/120 (84%) households completed follow-up. Household loss-to-follow up was 16%, primarily due to participants moving out of the study area, and did not vary significantly by trial arm (*p* = 0.47). There were 3/61 (5%) deaths in children <2 years in the intervention arm and 6/60 (10%) in the control (*p* = 0.28).

Among children <2 years, data were collected for 82% (1138/1382) of possible person-weeks of diarrhea. Baseline characteristics were distributed evenly between the trial arms, with the exception of mother's marital status, sex of child <2 years, and reported diarrhea (Table 1). Only 12% (14/121) of children <2 years were reported to be HIV-positive, 50% (61/121) were negative, and 38% (46/121) had not been tested by the end of our study.

**Table 1:** Selected baseline characteristics of intervention and control households.

		Intervention	Control
**Demographics**	Number of households	61 (51%)	59 (49%)
	Number of households in Ngwerere	27 (44%)	27 (46%)
	Number of households in Kasisi	34 (56%)	32 (54%)
	Number of people	299	300
	Number of children 6–12 months	61	60
	Median persons per household (range)	5 (2–10)	5 (2–10)
	Median mother's age (range)	28 (17–44)	30 (18–41)
	Mother is married or living with partner	54 (89%)	41 (69%)
	Mother has some education	49 (80%)	48 (81%)
	Mother is HIV-positive	51 (84%)	49 (83%)
	Mother on antiretroviral therapy	17 (28%)	16 (27%)
**Socioeconomic quintiles**	Lowest	3 (5%)	14 (24%)
	Low	4 (7%)	4 (7%)
	Middle	20 (33%)	13 (22%)
	High	21 (34%)	16 (27%)
	Highest	13 (21%)	12 (20%)
**Water Source**	Piped into home or yard	3 (5%)	3 (5%)
	Public standpipe	12 (20%)	10 (17%)
	Borehole	7 (11%)	11 (19%)
	Protected dug well	3 (5%)	7 (12%)
	Unprotected dug well	35 (57%)	27 (46%)
	Surface Water	1 (2%)	1 (2%)
**Water, Sanitation & Hygiene practices**	Report usually treating water	12 (20%)	13 (22%)
	Report usually chlorinating	12 (20%)	11 (19%)
	Report usually boiling	0 (0%)	3 (5%)
	Had treated water at time of visit	6 (10%)	7 (12%)
	Water storage container covered	54 (89%)	49 (83%)
	Use cup used to draw water from storage container	10 (16%)	17 (29%)
	Improved sanitation facility	15 (25%)	16 (28%)
	Soap present in household	27 (44%)	32 (54%)
**Water Quality**	Household TTC Geometric Mean (95%CI)^1^	272 (157–470)	317 (179–564)
	Source TTC: Geometric mean (95% CI)^1^	117 (72–190)	193 (114–328)
	Household free chlorine ≥0.2 mg/L^1^	1 (2%)	0 (0%)
	Source free chlorine ≥0.2 mg/L^1^	0 (0%)	0 (0%)
**Diarrhea in all household members**	Diarrhea in the past 7 days^1^	44 (15%)	27 (9%)
	Persistent diarrhea in the past 7 days^1^	10 (3%)	5 (2%)
	Persistent diarrhea in the past 7 days^1^	10 (3%)	5 (2%)
**Children <2 years**	Median age (SD) in months at recruitment	7.5 (1.9)	6.9 (1.9)
	Male	34 (56%)	22 (37%)
	Diarrhea in past 7 days	18 (30%)	17 (28%)
	Persistent diarrhea in past 7 days	4 (7%)	4 (7%)
	HIV-positive, if known	2 (3%)	1 (2%)
	Mean (SD) Weight-for-age z-score	−0.81 (1.42)	−0.97 (1.59)
	Currently breastfed	49 (80%)	46 (77%)
**Study Follow up Time**	Mean follow up (min-max) in months	11.2 (7–12)	11.3 (8–12)

1 Data are missing for 1 household on stored water TTC, 2 households on source water TTC, and 3 households on stored water chlorine residual. Three individuals are missing data on reported diarrhea and 5 individuals missing data on persistent diarrhea.

### Filter use

Most households used the filters throughout the study (Table 2). Households were classified as reported users in 96% (596/620) of all household visits and as confirmed users in 87% (540/620) visits. If we were to restrict our definition of confirmed user to only those that had at least 1 log_10_ removal of TTC, 82% (507/620) of intervention households would still be considered confirmed users. Among households that did not meet the criteria of confirmed users, 4% (24/620) visits had stored water of somewhat better water quality compared to unfiltered water (<1 log_10_) and therefore may have been actually using the filter. In instances when households did not have stored filtered water at the time of visit (3% of all visits, 16/622) the mother reported that she did not have time to filter the water. Only 3/61 (<5%) of filters had to be replaced during the study; 1 clogged and 2 were eaten by rats along the filter tubing

**Table 2:** Filter use and acceptability among intervention households.

	Final Visit		All Visits	
	N = 53	%	N = 627	%
**Filter Use**				
Reported user^1^	51/53	96%	596/620	96%
Confirmed user^2^	49/53	92%	540/620	87%
Exclusive use by mother today/yesterday^3^	49/53	92%	591/624	95%
Exclusive use by child <2 years today/yesterday^3^	48/50	96%	171/184	93%
Filter present in household	53/53	100%	625/626	>99%
Filtered water for drinking today or yesterday	53/53	100%	606/624	97%
Currently have filtered water stored^4^	51/53	96%	606/622	97%
Always used filter in past week	53/53	100%	620/623	>99%
Stored filtered ≥1 log_10_ TTC lower than unfiltered water, or <10 TTC/100 mL	49/51	96%	557/604	92%
Median volume of filtered water used per day (range)^5^	5 L (5 L)		5 L (2.5–20 L)	
Mother is responsible for filter	53/53	100%	617/626	99%
**What people like best about the filter**				
Provides safe water	40/53	75%	337/618	55%
Improves water taste	7/53	13%	129/618	21%
Provides good water	5/53	9%	143/618	23%
Easy to Use	1/53	2%	11/618	2%
**What people like least about the filter**				
Nothing – everything is ok	53/53	100%	615/621	99%
Flow rate is too slow	0/53	0%	3/621	<1%
Filter is broken/has a problem	0/53	0%	2/621	<1%
Doesn't provide enough water	0/53	0%	1/621	<1%
**Filter Maintenance** 6				
Backwashed today or yesterday	52/53	98%	601/624	96%
Cleaned pre-filter today or yesterday	52/53	98%	603/624	97%
**Water Storage**				
Using storage container provided	53/53	100%	623/625	>99%
Storage container capped	52/53	98%	623/624	>99%
Only store filtered water in supplied containers	51/53	96%	610/624	98%

1 Households were classified as “reported users” if 1) the filter was observed at the time of visit, 2) the storage vessel contained water reported to be treated, and 3) the respondent reported using the filter today or yesterday.

2 Households were classified as “confirmed users” if in addition to the criteria for reported users, there was at least a 1 log_10_ TTC improvement in stored household water over unfiltered water, or stored water quality was <10 TTC/100 ml.

3 Exclusive use was defined as not drinking any unfiltered water today or yesterday. For all households that did not report exclusive use, the reason for drinking unfiltered water was that they were away from home. For children <2 years, 3 children in intervention arm died so there are data missing at the final visit. Exclusive use for children <2 years data were only collected in the last quarter of the study period.

4 Mothers that didn't have filtered water reported that they did not have time to filter.

5 5 L is 1 container provided; all households reported 1 container (2 households missing data).

6 Households were instructed to backwash and clean the pre-filters daily, as recommended by the manufacturer.

Mothers reported exclusively using the filters in 95% (591/624) of all visits. For children <2 years, exclusive use was reported in 93% (171/184) of all visits. Reasons for not using the filter exclusively were that the mother or children were away from home, such as visiting relatives or at church. Almost all households (>99%, 623/625 visits) reported using the storage containers provided to store filtered water. Results at the final visit were similar to those at all visits (Table 2).

### Water Quality

**Figure 2:**
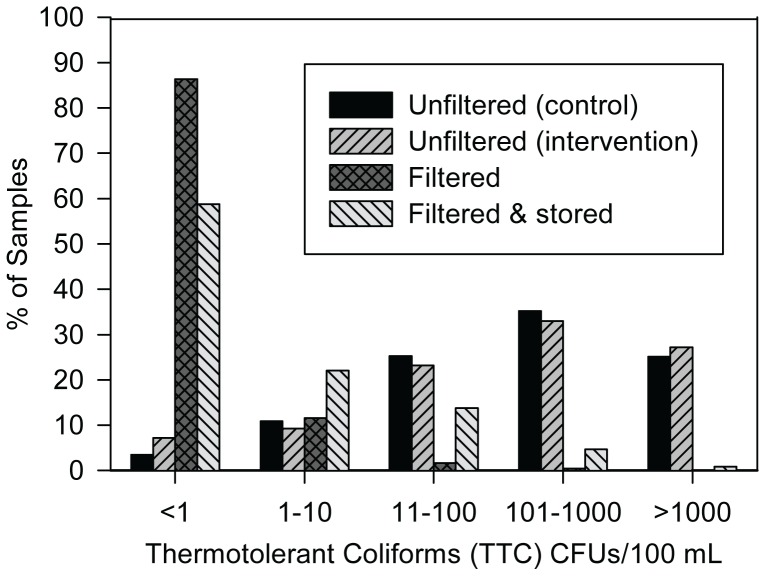
Water quality testing results. Unfiltered water is for all households; filtered and stored filtered is only for the intervention arm.

Unfiltered water had a geometric mean of 190 TTC/100 mL (95% CI: 147–245 TTC/100 mL), with 60.3% (720/1194) of samples over 100 TTC/100 mL (Figure 2). 3.3% of unfiltered intervention group water samples and 4.5% of unfiltered control group water samples yielded plates that were TNTC; no filtered samples and filtered and stored samples resulted in TNTC plates. Unfiltered water did not differ significantly between the intervention and control groups (geometric mean 199 vs. 181 TTC/100 mL, respectively, *p* = 0.61). In intervention households, water quality was significantly better in filtered water (geometric mean of 1.2 TTC/100 mL; 95% CI: 1.1–1.2 TTC/100 mL) and stored filtered water (geometric mean of 2.7 TTC/100 mL; 95% CI: 2.3–3.0 TTC/100 mL) compared with unfiltered water (Figure 2). The quality of stored drinking water was significantly better in intervention households than control households (geometric mean 3 vs. 181 TTC/100 mL, respectively, *p*<0.001). In intervention households, the geometric mean removal from influent (unfiltered) to effluent was 2.2 log_10_ TTC/100 mL (95% CI: 2.1–2.3 log_10_ TTC/100 mL), corresponding to a 99.4% (95% CI: 99.3–99.5%) reduction.

### Reported Diarrhea

**Figure 3:**
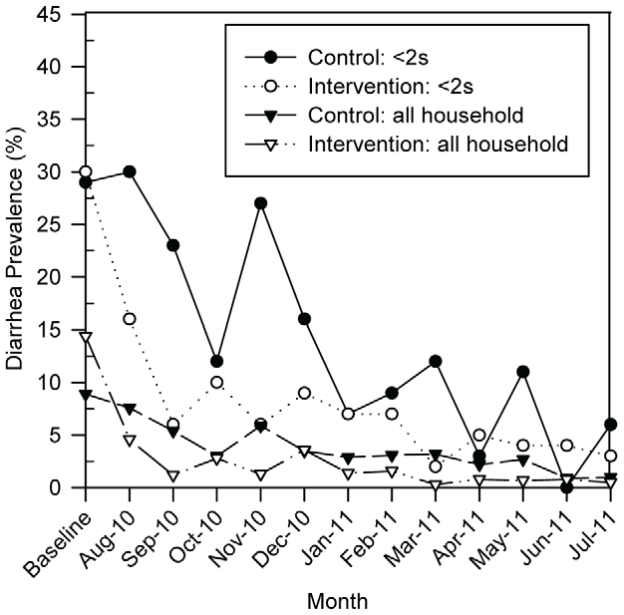
Longitudinal prevalence of diarrhea in children <2 years and all household members. Data for July 2010 are grouped with August 2010, due to follow-up visits commencing the final week of July.

Diarrhea longitudinal prevalence in children <2 years was 13.6% (72/530) in the control arm and 6.6% (40/608) in the intervention arm, representing a 53% reduction (longitudinal prevalence ratio, LPR = 0.47, 95% CI: 0.30–0.73, *p* = 0.001) (Table 3 and Figure 3). When restricted to children of HIV-positive mothers, the intervention was associated with a 50% reduction in diarrhea (LPR = 0.50, 95% CI: 0.31–0.80, *p* = 0.004). For all household members, diarrhea longitudinal prevalence was 3.5% (101/2906) in the control group and 1.6% (50/3168) in the intervention (LPR = 0.46, 95% CI: 0.30–0.70, *p*<0.001).

**Table 3:** Longitudinal prevalence of diarrhea in intervention and control groups.

	% Weeks with diarrhea of total possible person-weeks of diarrhea		LPR^1^ (95% CI)	P
	Intervention	Control		
**Diarrhea**				
<2 years	6.6% (40/608)	13.6% (72/530)	0.47 (0.30–0.73)	0.001
<2 years, HIV-exposed^2^	7.1% (36/509)	13.8% (58/419)	0.50 (0.31–0.80)	0.004
<5 years	4.3% (42/967)	8.9% (79/891)	0.51 (0.32–0.80)	0.003
All household	1.6% (50/3168)	3.5% (101/2906)	0.46 (0.30–0.70)	<0.001
**Persistent diarrhea (≥14 d)**				
<2 years	2.1% (13/608)	3.2% (17/529)	0.63 (0.28–1.40)	0.26
<2 years, HIV-exposed^2^	2.2% (11/509)	3.3% (14/419)	0.61 (0.25–1.49)	0.28
<5 years	1.5% (14/967)	1.9% (17/890)	0.77 (0.35–1.70)	0.51
All household	0.6% (18/3168)	0.7% (21/2904)	0.75 (0.37–1.53)	0.43

LPR = Longitudinal Prevalence Ratio.

1 Accounting for repeated measures (children <2 years) and clustering within household (all household data).

2 Child is considered HIV-exposed if their mother is HIV-positive.

Diarrhea was classified as persistent (≥14 days) in 26.2% (39/149) of reported weeks with diarrhea for all household members and 27.0% (30/111) of reported weeks with diarrhea for children <2 years (Table 3). Most persistent diarrhea occurred in children <2 years (76.9%, 30/39), and the 5 people who had more than one visit with persistent diarrhea were all children <2 years. The intervention resulted in reductions in persistent diarrhea for children <2 years (LPR = 0.63, 95% CI: 0.28–1.40, *p* = 0.26) and all household members (LPR = 0.75, 95% CI: 0.37–1.53, *p* = 0.43) though results were not statistically significant.

### Weight-for-age z-scores (WAZ) in children <2 years

There was no evidence of a difference between the intervention and control groups in mean WAZ scores (−1.21 vs. −1.24, respectively, *p* = 0.92). Adjusting for baseline WAZ did not change this conclusion (−1.18 vs. −1.31, respectively, *p* = 0.85).

Children with concurrent diarrhea had lower average WAZ scores compared to children without diarrhea (−1.46 vs. −1.20, respectively, *p*<0.001). After adjusting for WAZ at baseline, mean WAZ scores among children <2 years with diarrhea were 0.26 lower than in children without diarrhea (95% CI: −0.37 to −0.14, *p*<0.001).

### Water Quality, Diarrhea, and WAZ

**Figure 4:**
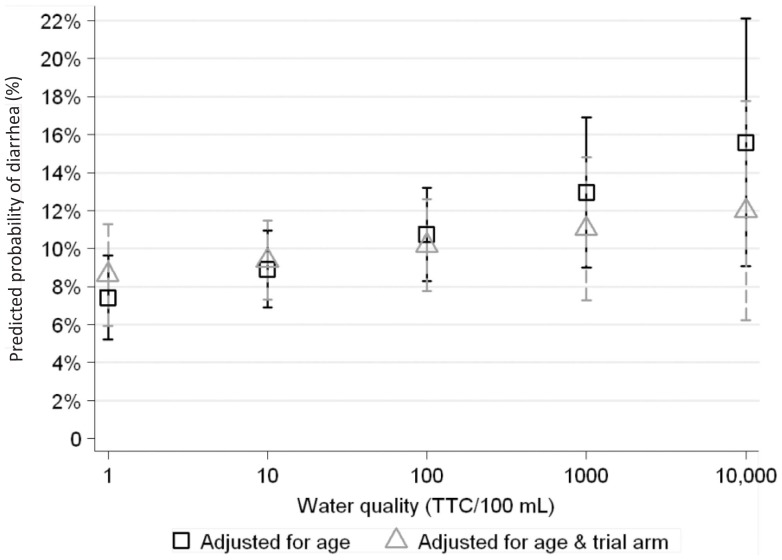
Water quality and diarrhea in children <2 years. Water quality is of stored drinking water (stored filtered water for intervention households and unfiltered water for control households). If intervention households did not have stored filtered water available, it was assumed they were drinking unfiltered water. Both analyses are adjusted for age; adjusting for trial arm is examined separately due to the partial collinearity between trial arm and water quality. Predicted probabilities of diarrhea are from unadjusted and adjusted binomial regression models with log link functions and robust standard errors with GEE to account for repeated measures. Error bars represent 95% confidence intervals. Unadjusted model coefficients: ln(diarrhea prevalence) = −1.25+0.186(log_10_TTC)+−0.0991(child's age). Adjusted model coefficients: ln(diarrhea prevalence) = −0.868+0.0825(log_10_ water quality)+−0.0990(child's age)+−0.506 (trial arm).

There was a suggestion of a positive trend between diarrhea prevalence and household fecal water contamination (Figure 4). The results of the fractional polynomial models showed that the linear model adequately described the relationship between log diarrhea prevalence and log_10_ TTC. This relationship was significant for all household members (age-adjusted LPR for the increase in prevalence with log_10_ TTC = 1.29, 95% CI: 1.14–1.45, *p*<0.001), and for children <2 years (age-adjusted LPR for log_10_ TTC = 1.20, 95% CI: 1.05–1.39, *p* = 0.01). Though adjusting for trial arm attenuated the association between water quality and diarrhea, there was still weak evidence of an effect (age- and arm-adjusted LPR for log_10_ TTC = 1.15, 95% CI: 0.99–1.33, *p* = 0.07 for all household members; age- and arm-adjusted LPR for log_10_ TTC = 1.09, 95% CI: 0.92–1.28, *p* = 0.33 for children <2 years). In contrast, there was no evidence of an association of water quality and WAZ (mean change in WAZ for log_10_ TTC = 0.00, 95% CI: −0.05 to 0.04, *p* = 0.93); adjusting for trial arm did not change this conclusion.

### Mortality of children <2 years

During the study, there were 9 deaths, all in children <2 years; 3/61 (5%) in the intervention arm and 6/60 (10%) in the control. The cause of death was recorded as reported by the primary caregiver. In the intervention arm, only one death was gastrointestinal (reported as diarrhea/vomiting); other deaths were from respiratory illness and consuming rat poison. In the control arm, deaths were potentially all gastrointestinal-related (diarrhea/vomiting, diarrhea/malnutrition [3 children], diarrhea/coughing, and malnutrition). All but one were children born to HIV-positive mothers, and two children were known to be HIV-positive. There was no evidence of an impact of the intervention on all-cause mortality among children <2 years (RR = 0.56; 95% CI: 0.13–2.37, *p* = 0.43), though the study was not designed to detect a difference in mortality as an outcome.

## Discussion

To our knowledge, this study is the first randomized controlled trial to examine a HWTS intervention among HIV-positive mothers with young children. Our findings suggest that the intervention was used correctly and consistently, was highly effective in improving drinking water quality, and was protective against diarrhea.

Filter use was particularly high in our study; households were using the filters in 96% of visits and use was further confirmed with water quality testing in 87% of visits. Some of the households that did not meet the water quality testing criterion for confirmed use may have been actually using the filter, but recontamination during storage prevented the criterion from being met. It is possible that repeated surveying contributed to increased use of the intervention [47]; some studies have lower uptake of HWTS when delivered programmatically rather than in research-driven efficacy trials such as this [48], [49]. However, there is some evidence that use is particularly high for filtration compared to other HWTS technologies [35], [50]. Previous studies of LifeStraw filters reported 68% use 8 months after distribution (Boisson et al 2010) and 83% use 2 months after distribution LifeStraw Family and LifeStraw personal filters combined) [51]. Furthermore, it is possible that use may be particularly high among HIV-positive mothers with young children because of increased concern and awareness of health; chlorination use has been found to be high among similar populations [25], [29].

A previous field trial of the LifeStraw Family filter in the Congo also reported high rates of use (76%) [38]. However, nearly all householders in that study (83% of adults and 95% of children <5 years) reported also drinking from other untreated sources, compared with only 5% of mothers and 7% of children <2 years in our trial. The large difference in exclusive use may be attributable to the fact that in the Congo trial households were advised to only use water directly from the filter and were not provided with safe storage containers, implying that safe storage containers may be essential to ensure exclusive use of HWTS. At the same time, there is little evidence that the practice of storing water after it is filtered adversely impacted drinking water quality in the home.

Diarrhea reductions in our study exceeded the 35–44% commonly found by HWTS [14], [15], [16]. Diarrhea reductions may have been particularly high among our population because of the increased risk of water-related pathogens in households with PLHIV [13], [52], [53] and the performance of the intervention in removing the full array of microbial pathogens. Furthermore, use and exclusive use was high among our population, and there is an increased health impact among high-frequency HWTS users [28], [54]. However, the intervention did not result in significant reductions in persistent diarrhea among children <2 years or all household members. Previous research has found that household water treatment may be more effective in reducing shorter episodes of diarrhea compared to persistent diarrhea [55].

Water quality showed a positive trend with reported diarrhea, both for children <2 years and all household members. Interventions that improve water quality are known to reduce diarrheal disease [15], [56], though the relationship between drinking water quality bacterial indicators and general diarrheal disease is not well established [56], [57], [58]. An observational study in Tanzania found a relationship between health and fecal contamination on hands but not in stored drinking water [59], though a previous trial of a household ceramic filter in Colombia found a significant relationship between water quality and diarrhea [60]. In our study, the suggestion of positive trend between diarrhea and water quality supports our finding that the water quality intervention resulted in a reduction in diarrheal disease; presumably participants would be unable to base reported diarrhea on actual TTC levels in their water considering they were not aware of exact TTC levels.

Though we did not find an impact of the intervention on WAZ, we did detect a significant association between WAZ and reported diarrhea. The lack of difference in WAZ between our trial arms despite the reduction in reported diarrhea and the association between WAZ and reported diarrhea merits further discussion. It is possible that reported diarrhea data may be of questionable reliability; open trial designs of self-reported outcomes are subject to bias [61]. We cannot entirely rule out or assess the effects of biased self-reporting of diarrhea. However, the relationship between diarrhea and water quality is well-established and is the basis for international drinking water quality standards [62]. The fact that we observed this same relationship here suggests that our results are not solely attributable to bias self-report. Moreover, we found no association between WAZ and water quality; given that the intervention may only influence WAZ via water quality, the intervention may not be appropriate to improve WAZ. Furthermore, diarrhea and WAZ may be associated primarily due to persistent diarrhea. We did not find a significant reduction in persistent diarrhea in children <2 years (*p* = 0.26) and a previous trial in Guatemala found that a HWTS intervention mostly prevented short episodes [55], [63]. Therefore, the diarrhea experienced by our intervention arm may have been more persistent compared to the intervention group. This is supported by a stronger relationship between diarrhea and WAZ in the intervention arm than in the control arm (*p* = 0.003 for interaction); persistent diarrhea is known to impair growth [42], [64]. Though we cannot entirely discount the possibility of reporting bias, WAZ may not be an appropriate measure for diarrhea in HWTS trials, though further investigation is needed.

There are some limitations to our study. First, the reliance on self-reported data for diarrhea disease in a non-blinded HWTS intervention trial has previously been criticized [34], [35]. However, the suggestion of positive trend between water quality and diarrhea suggests that most of the self-reported diarrhea may be verifiable. Second, baseline diarrhea prevalence was not evenly distributed between our trial arms for all household members, though this would only result in a conservative estimate of the intervention effect and baseline diarrhea may not be predictive of diarrhea during the intervention period [63]. Third, because we recruited from health clinics, we were not capturing the most vulnerable population that does not have access to health facilities or is too sick to access these services. Finally, our study was conducted in Chongwe District, Zambia and may not be generalizable to other locations with different water quality and practices.

Despite these limitations, our findings indicate that HWTS may be particularly beneficial among HIV-positive mothers with young children. Though our study was not designed to examine mortality of children <2 years, our study results and previous research [65] suggest that HWTS may have the potential to reduce mortality in young children. The effect of HWTS on mortality of young children needs to be further explored in the form of a full randomized, controlled trial.

## Supporting Information

Protocol S1 **Trial Protocol.**
(DOC)

Checklist S1 **CONSORT Checklist.**
(DOC)
