# Supplementary material for: Assessing Water Filtration and Safe Storage in Households with Young Children of HIV-Positive Mothers: A Randomized, Controlled Trial in Zambia
Source: PLoS One. 2012 Oct 17;7(10):e46548. doi: 10.1371/journal.pone.0046548 (PMC3474778; doi:10.1371/journal.pone.0046548)
Supplement: Protocol S1 — Trial Protocol. (DOC) [file pone.0046548.s001.doc]

Assessing Water Filtration and Safe Storage in Households with Young Children of HIV-Positive Mothers: A Randomized, Controlled Trial in Zambia

Study Protocol

### 1. Background

Contaminated drinking water is a leading cause of morbidity and mortality in low-income settings. Safe drinking water is of particular concern for HIV-positive mothers since many HIV-infected Zambian women choose replacement feeding and early cessation of breastfeeding of infants to minimize the risk of transmission of the virus (CIGNIS study results; S Filteau, L Kasonka et al., submitted). In resource-poor settings, HIV-positive mothers must choose between breastfeeding their infant and potentially transmitting HIV, or formula feeding their infant and thereby risking diarrhoeal disease morbidity and mortality. However, the extent to which the drinking water actually being used by this population is safe, and what steps could be taken to ensure the microbiological quality of such water, is largely unknown.

Our previous work in Zambia found that HIV-positive mothers with children <2 years would potentially benefit from an intervention that encourages HIV-positive mothers to treat their water at the household level before using it for their young children and all household members. In a cross-sectional study of 254 households, we found that 26% of children <2 years had diarrhoea in the past week, 53% of children <2 years received supplemental water in the past 2 days, and 70% of households had faecally contaminated drinking water. This proposed study aims to assess a household water treatment technology in the form of a randomized controlled, trial.

**2. Objectives**

This randomized, controlled trial (RCT) pilots a household water treatment and safe storage (HWTS) technology, the LifeStraw Family filter. This research aims to answer the following question:

*Would children <2 years and other members of households in which HIV-positive mothers are providing replacement and complementary feeding potentially benefit from the use of a filter designed to eliminate microbial pathogens from drinking water at the household level?*

The specific objectives of the pilot study are:

- To examine the use, longevity and acceptability of LifeStraw Family, both for children <2 years and for drinking water by all household members
- To examine the microbiological performance of LifeStraw Family, measured in terms of thermotolerant coliforms (TTC), a well-established WHO indicator organism for faecal contamination (WHO 2004)
- To assess the impact of the intervention on the longitudinal prevalence of diarrhoea among children < 2 years and all household members, measured both as reported by the primary caretaker of the child and by their weight-for-age z-score (WAZ)—a proxy for diarrhoea (Schmidt, Boisson et al. 2009).

**3. Sample Size**

One hundred and twenty households will be followed for one year. The sample size of one-hundred households is based on the primary outcome (adoption) and is based on an estimated 60% of the study population satisfying the conditions for use, with a standard of 0.05, for a 95% confidence interval of 50-70%. An additional 20 households of HIV-negative mothers will be included to reduce stigma of the study. The study population will be followed for one year to account for seasonal variability in water quality.

**4. Location**

The site(s) will be in or around Lusaka, Zambia and should meet the following criteria:

- Sufficient number of households with children <2 years born to HIV-positive mothers who know (or willing to be tested) and are willing to disclose their HIV status for purposes of this study
- Available testing, care, and counselling for HIV-positive individuals
- Active health clinic with staff that can help with the recruitment process, through ART programs, nutrition programs, under-five clinics, etc
- Population relying on unsafe drinking water, characterized by unimproved water supplies and thermotolerant coliform (TTC) contamination, as measured in CFUs/100 mL
- Majority of households are not treating their water in some microbiologically effective manner (e.g., boiling)
- The population has sufficient quantity and access to water supplies, so that major water deficiency is microbiological quality
- The population and community leaders desire to participate in the study

**5. Study Population and Recruitment**

Study participants will be recruited through the community health clinic for the catchment area or areas. In our cross-sectional study, it was particularly efficient to recruit through ART programs, nutrition programs, and the under-five clinics at the community health centres; depending on the health centre(s) chosen, we may use similar recruitment methods. Women will be eligible to participate in the study if (i) they report that they are HIV-positive based on clinical testing, (ii) they currently have a child age six months to one year at the beginning of the study, and (iii) they reside in a household located in the designated peri-urban area(s) in or around Lusaka, Zambia, (vi) drinking water of poor quality. HIV-status of mothers will be confirmed by the child’s exposure status (‘exposed’ or ‘unexposed’) on the child’s health record. Women that are interested in participating will be asked for consent and household location information will be obtained for follow-up. It is possible that some of the same HIV-positive mothers from our cross-sectional study will be included in the RCT. In order to reduce stigma, we plan to include an additional 20 HIV-negative mothers (10 in intervention group and 10 in control). These mothers will also be recruited from the selected community health clinic(s). We will be asking about infant HIV-status if known, though this will not be required for participation. Participating mothers will be reminded that HIV-testing of children is available in Zambia free-of-cost, including PCR testing from 6 weeks. If mothers subsequently choose to have their children undergo HIV testing, we will ask that they contact us with the results.

### 6. Baseline Survey and Intervention

*Baseline survey and randomization*

Before the water filter is distributed, a baseline survey will be conducted of the recruited participants. The baseline study will include information on demographics, sanitation facilities, hygiene practices, water sources and treatment practices and other factors that could potentially impact the outcomes of interest. After the baseline survey, the intervention will be randomly allocated to half of the recruited participants. Participating households will be numbered consecutively and then allocated into the two groups using a random number generator. Households that do not receive the filter at the beginning of the study will receive the filter at the end of the study. Households will receive training on how to use and maintain the filter prior to receiving the intervention.

*The Intervention*

The intervention consists of the Lifestraw Family filter for household water treatment and a storage container to encourage safe water storage of treated water. The LifeStraw Family is a HWTS filtration technology shown to remove microbial contamination from drinking water and to be acceptable to users. The device was chosen because it has been shown to be effective against the full array of microbial pathogens and to have a longevity that exceeds one year (Clasen, Naranjo et al. 2009). Developed by Vestergaard Fransen SA, LifeStraw Family is a new household-based gravity filter system that uses ultrafiltration mechanisms to remove pathogens. The filtration unit consists of a hollow-fibre cartridge of 20 nm diameter pore size. Untreated water is poured into a 2.5L container, flows down a 1m long tube designed to provide head pressure, and through the ultrafiltration cartridge where it is dispensed via tap. The cartridge is also equipped with a backwash inlet to facilitate cleaning of the cartridge, thus minimizing clogging and improving longevity. The flow rate of the device averages at 8.8L/hour (over design life of 18,000L with periodic cleaning and backwashing),(Clasen, Naranjo et al. 2009) and the flow shuts down when the hollow-fibres are exhausted, indicating when to replace the cartridge.

Independent laboratory testing of the filter has demonstrated high microbiological performance on the full array of waterborne microbes. Through 20,000L (111% of design life) at moderate turbidity (15 NTU), the technology achieved log_10_ reduction values of 6.9 for *E. Coli*, 4.7 for MS2 coliphage (proxy for enteric pathogenic viruses), and 3.6 for *Cryptosporidium* oocysts (Clasen, Naranjo et al. 2009). Testing confirmed that the device meets the highest international standards for microbiological water purifiers. The device has the advantage (compared to chlorination technologies) of removing protozoan cysts such as *Cryptosporidium* oocysts, which are particularly prevalent in malnourished or HIV-positive infants in Africa (Mor and Tzipori 2008). It also removes viruses, a major challenge for most gravity-based filter medium, such as ceramics. Since the device is relatively easy to use, improves the appearance of water and does not rely on chemicals that adversely impact taste, adoption and use are likely to be greater than other HWTS products. Moreover, with a 20,000L design life, the unit can provide protection for an entire household for up to four years, minimizing supply chain challenges that present obstacles for consumable water treatment products.

The intervention will also include a vessel for safely storing water following treatment. Previous fieldwork with the LifeStraw Family filter has suggested that exclusive use of water treated with the filter is less then optimal, especially among young children, because of the inability to safely store and conveniently access water after treatment. The storage device will be procured locally and piloted to ensure acceptability by the study population. At a minimum, it will contain an opening that is too small to permit the entry of hands, and will allow drawing of water only via a tap or by pouring.

**7. Research Methods and Outcomes**

Each of the 120 households will be visited monthly for water quality testing and household questionnaires. Specifically, the research methods will include:

- Household surveys, observations, and focus group discussions to evaluate use, longevity, and acceptability (objective 1)
- Routine water quality testing of source, treated, and stored water to evaluate microbiological performance (objective 2)
- Regular disease surveillance, including household visits to assess reported diarrhoea, changes in weight and hospital admissions for diarrhoea (objective 3)

*Use, Longevity and Acceptability*

Previous research has suggested that a principal reason for failure to achieve the potential health gains from HWTS interventions is sub-optimal adoption—particularly exclusive use by the most vulnerable segments of the population. In this RCT, use will be the principal outcome. Each filter unit will be encoded with a number and the date distributed will be recorded. The investigator will develop criteria for assessing use and a set of indicators based thereon (e.g., filter mounted for use in the household, filter reported to have been used on the day of the visit or the previous day, storage vessel contains water that is reported to have been treated, and microbiological quality of water is at least 1 log lower in TTC than source water). Thereafter, the investigator will determine whether the device is in use during each household visit using the specified indicators that combine reporting and observations. Furthermore, approximately 10% of the filters will include flow meters that record the quantity of water that has been run through the filter to objectively measure filter use. If the device is not being used at all, the investigator will record the reasons according to the mother. If the device is no longer in use due to clogging, the investigator will record the date it clogged and attempt to restore use by normal cleaning. If service cannot be restored, the investigator will replace the cartridge or other failed component and note the change.

Information will also be gathered on use of filtered water to make replacement feeds for young children through household questionnaires and observations. Mothers will be asked during household visits about the water given to young children directly or added to other drinks or food consumed by young children. Observations may be included on approximately 20%-50% of households to examine water practices concerning infant feeding.

The acceptability of the technology will be evaluated by conducting household surveys, household observations, and focus group discussions (FGDs). Households will be interviewed monthly through household questionnaires, and additional information will be gathered on acceptability after approximately 4 and 8 months. Mothers will be asked to participate in FGDs at the end of the study to provide feedback on the technology including perceived benefits and limitations.

On two occasions during the course of the study, about a quarter of the households will be visited by investigators to obtain more extensive information about providing children <2 years food and water. During these visits, we will be talking with the primary caretaker of the child and will also be asking her to demonstrate certain practices and allowing the investigator to spend time in the home observing the practices directly. Particularly, observations will explore exposure pathways for contaminated water, and for the intervention households, to what extent the filtered water is given to young children.

*Microbiological Performance*

The performance of the HWTS technology will be evaluated by bacteriological water quality testing. Water samples will be taken during unannounced household monthly visits of all households. For households that have not received the filter, water samples will be collected of i) source water and ii) stored drinking water. For households that have received the filter, water samples will be collected of i) source water (influent water), ii) effluent water out of the filter, and iii) stored drinking water. Source drinking water will be collected to determine technology performance by comparing effluent and influent water quality. For the stored drinking water, the householder will be asked if there is any drinking water in the house at that moment and samples will be collected from the vessel that the householder identifies as being used at that moment to fill a cup or glass for drinking.

All samples for analysis will be collected in sterile Whirl-Pak™ Bags containing a tablet of sodium thiosulfate to neutralize any disinfectant. Samples will be placed on ice and processed within 4 hours of collection to assess levels of thermotolerant (faecal) coliforms. Microbiological assessment will be performed using a DelAgua field incubator (University of Surrey) in accordance with the Standard Methods (Eaton, Franson et al. 2005).

*Diarrhoea Prevalence*

This study is designed to use two techniques on diarrhoeal surveillance: i) conventional report of diarrhoea prevalence and ii) a novel technique using weight-for-age z-scores (WAZ) as a proxy for diarrhoea. Diarrhoea prevalence will be recorded for the children <2 years and for all household members as longitudinal prevalence from 7-day recalls by the mothers. Diarrhoea will also be objectively assessed by recording hospital admissions for diarrheal disease. Longitudinal prevalence (the proportion of days with diarrhoea divided by the number of days under observation) is a preferred metric for assessing diarrhoea because it is a strong predictor of long-term health outcomes (Morris, Cousens et al. 1996). In the cross-sectional study, it was determined that a 7-day recall was appropriate for the study population. Diarrhoea will either be defined according to the WHO definition of 3 or more loose stools within a 24-hour period (WHO 2005), or using a local definition if found to be comparable. Diarrhoea will be assessed during household visits approximately every month, totalling 12 visits over the course of one year. Feeding practices for the children <2 years will be included in household questionnaires which may affect diarrhoea prevalence.

A novel approach for measuring diarrhoea is to use weight-for-age z-scores (WAZ) as proxy (Schmidt, Boisson et al. 2009). Objective markers for diarrhoea are needed to avoid potential bias that may occur during in conventional self-report measurements. Short-term changes in WAZ have been found to be strongly associated with diarrhoea prevalence. In this study, we will pilot using WAZ to measure the effect of the intervention. Children <2 years will be weighed using baby scales during monthly visits, and date of birth will be verified on the child’s health card to calculate WAZ.

### 8. Data Analysis

Specific endpoints will be analysed corresponding to each objective.

| Objective #1 | - Percent of households using the filters during the entire study - Percent of households using the filters for children <2 years - Percent of households that had technical problems with the filters (clogging or breaking) - Qualitative feedback on filter acceptability |
| --- | --- |
| Objective #2 | - Median and range of TTC concentration in coliform forming units (CFUs) per 100 mL, of   - Source water   - Filtered drinking water   - Stored drinking water - Mean and range percent removal of TTC by LifeStraw Family |
| Objective #3 | - Relative risks of reported diarrheal prevalence and diarrheal hospital admissions in children < 2 and all household members, comparing intervention and control - Weight gain over 12 months of the study, using WAZ as a proxy for diarrhoea |

Survey data will be collected with paper questionnaires, entered into EpiInfo, and analysed using Stata10. Water quality data will be entered into Excel and analysed using Stata10. The statistical analysis will account for clustering by households as necessary, and intracluster correlation of repeated measurements on the same individual (Hayes and Moulton 2009). A logistical regression model will be fit and a multivariable analysis will adjust the model for potential confounders (age, socioeconomic status, etc) to compare intervention and control populations.

After study completion, a summary of the results will be communicated to all study participants at a meeting called for that purpose. Study participants in the control group will receive a LifeStraw Family filter. A full research report detailing the results of the investigation will be distributed to the ethics committees.

## 9. Ethics

This study will be submitted to the Ethics Committee of London School of Hygiene and Tropical Medicine (LSHTM) and to the Ethics Committee of University of Zambia. Informed, written consent will be obtained from all participants. Measures will be taken to ensure confidentiality for all participants and that the participants do not experience any additional hazards from this study. If participants are found to be in need of health care during the household visits, they will be referred to health clinics.

**10. References**

Clasen, T., J. Naranjo, et al. (2009). "Laboratory assessment of a gravity-fed ultrafiltration water treatment device designed for household use in low-income settings." Am J Trop Med Hyg **80**(5): 819-823.

Eaton, A. D., M. A. H. Franson, et al. (2005). Standard methods for the examination of water & wastewater, American Public Health Association, American Water Works Association, Water Environment Federation,.

Hayes, R. J. and L. H. Moulton (2009). Cluster Randomised Trials. London, CRC Press.

Kelly, P., K. S. Baboo, et al. (1997). "Cryptosporidiosis in adults in Lusaka, Zambia, and its relationship to oocyst contamination of drinking water." J Infect Dis **176**(4): 1120-1123.

Kelly, P., J. Todd, et al. (2009). "Susceptibility to intestinal infection and diarrhoea in Zambian adults in relation to HIV status and CD4 count." BMC Gastroenterol **9**: 7.

Mor, S. M. and S. Tzipori (2008). "Cryptosporidiosis in children in sub-Saharan Africa: A lingering challenge." Clinical Infectious Diseases **47**(7): 915-921.

Morris, S. S., S. N. Cousens, et al. (1996). "Is prevalence of diarrhea a better predictor of subsequent mortality and weight gain than diarrhea incidence?" Am J Epidemiol **144**(6): 582-588.

Schmidt, W.-P., S. Boisson, et al. (2009). "Weight-for-age z-score as a proxy marker for diarrhoea in epidemiological studies." JECH Online FirstBMJ Publishing Group Ltd( 10.1136/jech.2009.099721 ).

WHO (2004). Guidelines for Drinking-water Quality, Third Edition. Geneva, World Health Organization.

WHO (2005). The Treatment of diarrhoea : a manual for physicians and other senior health workers -- 4th rev. Geneva, Department of Child and Adolescent Health Development.
